# Editorial: The Immunological Implications of the Hygiene Hypothesis

**DOI:** 10.3389/fimmu.2021.732127

**Published:** 2021-08-17

**Authors:** Petra Ina Pfefferle, Idoia Postigo, Holger Garn

**Affiliations:** ^1^Medical Faculty, Comprehensive Biobank Marburg (CBBMR), Member of the German Biobank Alliance (GBA) and the German Center for Lung Research (DZL), Philipps University of Marburg, Marburg, Germany; ^2^Faculty of Pharmacy and Lascaray Research Center, Parasitology and Allergy Research Group, University of The Basque Country (UPV/EHU), Vitoria, Spain; ^3^Medical Faculty, Biochemical Pharmacological Center (BPC), Translational Inflammation Research Division & Core Facility for Single Cell Multiomics, Member of the German Center for Lung Research (DZL), Philipps University of Marburg, Marburg, Germany

**Keywords:** hygiene hypothesis, inflammation, microbiome, bacteria, parasites, environment

Human beings developed in close vicinity to their natural environment during their long-lasting history. During this time, a stable relationship was established between the highly diverse environmental influences and physiological processes in the human body resulting in an optimal adaptation of humans to the respective ecological niches. Thereby, environmental factors involved both external impacts including frequent (early-life) microbial or parasitic infections and the establishment of an internal microbial community, the microbiome. Due to urbanization and industrialization these relationships were massively disturbed and new interactions developed. Very recently, this was impressively shown again by Wibowo et al., who reconstructed ancient microbial genomes from 1.000 – 2.000 year-old stool samples and showed that these paleofecal microbiomes are similar to those from people currently living in non-industrialized areas of the world, but are significantly different from microbiomes in humans living in highly industrialized countries (1). These rather recent changes are considered to have substantially contributed to the development of modern civilization disorders, which predominantly involve non-communicable diseases (NCDs) such as allergies, autoimmune and neurological diseases, and cancer. Contemplating the potential inverse relationship of early-life exposure to a highly diverse microbial environment and the prevalence of NCDs, the Hygiene Hypothesis was established about 30 years ago. Since then, several studies have added new evidence and refined the postulate justifying this Research Topic on immunological implications of the Hygiene Hypothesis, which now constitutes an impactful collection of review and original articles addressing recent developments and novel findings combined with retrospective and prospective views in this still highly topical field of scientific research.

First, von Mutius, one of the most prominent key opinion leaders in the field provides a concise personal statement discussing the protective effects of traditional farm exposures on the development of childhood asthma and allergies. From her epidemiological point-of-view, (microbial) diversity in the farm environment is key for its allergy-preventive effects (2) and this is supported by the fact that no single component has so far been identified that confers protection. However, two key elements of the protective farm effect have been described, exposure to animal sheds, in particular to cowshed dust, and the consumption of unprocessed cow’s milk. Moreover, pregnancy has been recognized as an early window of opportunity to prevent immune deviation and allergic outcomes in offspring by traditional farm exposure implying a role for epigenetic imprinting. Finally it has been concluded that a multiplicity of exposures, *via* diverse routes, influence microbiome composition at different mucosal sites and overall these impacts are associated with a lower risk of asthma in farm populations. In a subsequent mini review, we provide a short chronological overview on landmark findings that developed and further shaped the Hygiene Hypothesis over time. We discuss microbial and nutritional aspects with regard to their association to the hypothesis and provide insights into our current understanding on underlying immunological mechanisms (Pfefferle et al.). While the Hygiene Hypothesis was originally established in the context of allergic diseases, Jean-Francois Bach expanded its application to other conditions such as autoimmune diseases in the early 2000s (3). In this Research Topic, he contributes a sophisticated review article revisiting the Hygiene Hypothesis in the context of autoimmune disorders. Similar to allergies, these diseases represent complex multifactorial and polygenic chronic conditions and thus the underlying mechanisms of disease initiation and perpetuation are multiple and complex as well. This review summarizes the current epidemiological evidence for the inverse association of infections and autoimmune disorders and provides insights into the current immunological mechanisms thought to be involved in these interactions (Bach). Expanding the focus towards a more holistic view, Fiuza et al. present their concept of considering humans and their entire associated microbial communities as evolutionarily developed holobionts. They discuss the interactions between the entirety of human-associated microbial communities and the developing immune system during ontogenesis, and how shifts in environmental conditions may impact these well-balanced interrelationships. Further, in line with the Hygiene Hypothesis, the authors describe epigenetic modifications as fundamental mechanisms in gene-environment-interactions and as driving forces for the maturation of the immune system.

A better understanding of the mechanisms underlying the Hygiene Hypothesis may lead to new concepts of allergy prevention. In this context, Sarate et al. demonstrate that exposure of conventional or gnotobiotic mother mice with the probiotic bacterium *Escherichia coli* Nissle 197 may decrease polysensitization to birch and grass pollen allergens and allergic inflammation in the offspring. Interestingly, recombinant variants of the bacterial strain expressing birch and grass pollen allergens did not result in the same extent of allergy prevention. The review by von Mutius already pointed to ß-lactoglobulin (BLG) as an abundant component in raw cow’s milk and the ambient air of cowsheds. Based on these findings, Afifi et al. hypothesized that BLG may exert tolerogenic effects thereby contributing to the allergy-preventiving farm effects. Using a mouse model, they were able to show that prophylactic pretreatment with the ligand-filled holoBLG, but not the empty apoBLG, reduced allergen-specific antibody titers, features of antigen presentation, and allergic T cell responses, in allergen-specific (towards apoBLG) as well as an allergen-non-specific (towards birch pollen allergen) manner. In an ambitious latent class analysis (LCA) approach, Hose et al. analyzed daily food consumption patterns in relation to the gut microbiome of children by one year of age and at school age (time point of asthma initiation). The authors found excessive meat consumption to be associated with later asthma development while consumption of milk products compensated for such an effect. Interestingly, microbiome analyses indicated that this association might be related to the “iron battle field” in the gut. Free iron is a limiting nutritional factor for the metabolism of both the host and bacteria. Unbalanced meat consumption fosters accumulation of gut-associated bacterial strains equipped with siderophores that are highly capable of taking up free iron from the gut lumen. Since milk products in general, and unprocessed milk in particular, contain large amounts of iron-binding beta-lactoglobulin, this milk compound might effectively combat the growth of such bacterial iron competitors.

The balance of the local microbiological environment in mucosal tissues is tightly controlled by a variety of innate and adaptive defense systems. Among them, antimicrobial peptides such as the human β-defensin 2 (hBD-2) play a key role and thus disturbances in their production may favor the development of allergic and/or autoimmune disorders. In their manuscript, Borchers et al. clearly demonstrate that genetic variations associated with insufficient hBD-2 production may represent a risk factor for the development of such diseases. Accordingly, prophylactic administration of hBD-2 was able to reduce pulmonary inflammation and improve lung function parameters in a mouse model of allergic airway inflammation. In the context of gene-environment-interactions, genetic predisposition may also represent a decisive factor in shaping early immune mechanisms in the presence of parasitic infection and in asthma development. In a genome-wide association study (GWAS) on *Ascaris lumbricoides*-infected children, Carneiro et al. associate *WSB1* and *IL21R* genetic variants with markers of type-2 immune responses. *WSB1* but not *IL21R* gene expression was suppressed in infected children and increased methylation was concomitantly observed in the *WSB1* promoter region. The WSB1/IL21R pathways may thus represent potential targets for the treatment of type-2-mediated diseases. In the same research area, Korb et al. present an experimental approach that underlines the role of glycosylation of parasite-derived extracts in anti-allergic immunomodulation. Using an ovalbumin-model of allergic airway inflammation, in which sensitized mice were intranasally treated with native *Toxoplasma gondii* lysates, they observed attenuation of experimental asthma parameters. In contrast, application of deglycosylated lysates was shown to exert no protective effect indicating that the carbohydrate modifications are relevant for the strong anti-inflammatory effects provided by *T. gondii*-derived extracts. The potential role of sugars and related receptors in the regulation of immune mechanisms are further highlighted in a mini review provided by Peters and Peters who emphasize the role of carbohydrate lectin receptors (CLRs) in allergic airway inflammation. Diverse allergen glyco-epitopes may bind to such CLRs, thereby facilitating their uptake and presentation. However, CLRs have also been shown to mediate inhibitory signals and a number of studies have demonstrated anti-inflammatory effects after binding carbohydrate ligands of microbial origin. The authors discuss the involvement of sugar moieties in immune regulation and how CLRs and their ligands may contribute to allergy and asthma protection.

This Research Topic is completed by a forward-looking mini-review by Garn et al. focusing on new developments and insights in immunological and allergy research and how these current perceptions further finetune our understanding on mechanisms underlying the Hygiene Hypothesis. Finally, the application of the Hygiene Hypothesis to explain the increasing incidence of conditions other than allergies and autoimmune disease is also discussed, underscoring the enduring importance of this scientific concept.

## Author Contributions

All authors contributed to the article and approved the submitted version.

## Conflict of Interest

The authors declare that the research was conducted in the absence of any commercial or financial relationships that could be construed as a potential conflict of interest.

## Publisher’s Note

All claims expressed in this article are solely those of the authors and do not necessarily represent those of their affiliated organizations, or those of the publisher, the editors and the reviewers. Any product that may be evaluated in this article, or claim that may be made by its manufacturer, is not guaranteed or endorsed by the publisher.
